# Biomimetic Functional Fluorinated Oxygen-Containing Coatings on 3D-Printing Composite Polymer Items

**DOI:** 10.3390/polym17182490

**Published:** 2025-09-15

**Authors:** Georgy Rytikov, Fedor Doronin, Andrey Evdokimov, Mikhail Savel’ev, Yuriy Rudyak, Victor Nazarov

**Affiliations:** Faculty of Printing Industry, Moscow Polytechnic University, 107023 Moscow, Russiarudyak@mail.ru (Y.R.);

**Keywords:** biomimetics, composite filaments, shark skin, tribology, wear, bulk modification, fluorination, additive manufacturing, polymer

## Abstract

We manufactured the 3D-printed prototypes with increased wear resistance using a combination of the following: biomimetic design (the shark skin was used as a natural object to follow), 3D-printing technological parameter regulation, rational choice of polymer matrix, dispersed filling ingredients and items’ surface gas-phase modification technique. It was established that the bulk modification of the PETG filament with montmorillonite, graphite nano-plates, and other ingredients can reduce the 3D-printed prototypes’ wear by up to eight times. The gas-phase fluorination of the product’s surface provides a decrease in the rest friction coefficient and temperature in the “indentor-3D-printed disk” contact pair. We obtained the texture models and quantified the degree of similarity between the shark skin and the 3D-printed prototypes’ surfaces.

## 1. Introduction

The improving of polymer materials made the items’ functional and operational properties an urgent and practically significant task of modern materials science [1,2,3]. The possibility and the prospects of purposefully forming biomimetic structures (designed to control the surface properties of different materials-made goods) were reported in [4,5,6]. The widely known effects of “lotus petals”, “butterfly wings”, “rice leaves” and “shark skin” were discussed in [7,8,9,10].

It is known [11,12,13,14] that shark skin’s unique hydrotribological characteristics are largely determined by the physical structure of the epithelial tissues of this fish [15,16]. Consequently, shark skin texture imitation can lead to the improvement of polymer materials-made products’ tribological characteristics [17,18,19]. The practical implementation of this concept requires appropriate 3D-design development, materials choice, and 3D-printing technological solutions rationalization [20,21,22].

The quantitative analysis of multiscale biological objects’ images [23,24,25] makes it possible to create the digital models of their surfaces and, as a result, to prototype [26,27,28] the functional coatings being developed at the macroscopic level using computer-aided design (CAD) systems. At the same time, it was noted in [29,30,31,32,33] that the quality of nature-like polymer materials items’ properties forecasting largely depends on the technique and accuracy of the imitated natural object’s structure (the original layout) and computer data analysis.

The meso-level directional texture regulation can be implemented using bulk modification techniques with the rationally selected fillers [34,35,36]. And the composite materials are often formed as a result of various ingredients incorporation into the initial polymer matrices [37,38,39]. It allows for direct transformation of not only the structural but also the functional characteristics of the manufactured products [40,41,42] (including bearings, brakes, bushings, gears, filled thermoplastics based sealing elements, etc. [43,44,45,46,47]).

The common polymer matrices’ bulk modification ingredients are the powders of aluminum oxide, copper, and molybdenum disulfide [48,49,50]. They reduce the friction coefficient and, as a result, increase the manufactured goods’ wear resistance [51,52,53]. And the composite polymer materials, as a rule, demonstrate high tribological efficiency in “composite-metal” and “composite-polymer” contact pairs [54,55,56].

The filled filament rheological characteristics change statistically significantly due to the bulk modification outlined in the materials’ elemental composition and chemical structure transformations. It leads to the need of the extrusion technological parameters’ correction [57,58,59]. In particular, when adjusting the product 3D-model slicing parameters [60,61,62], it is necessary to establish the actual value of the melt flow index (MFI) and determine the optimal extrusion temperature for each new composite filament. The corresponding procedures should be carried out using standardized industrial plastometers according to the current standards (ASTM/GOST). But they are often inaccessible to the fused filament fabrication (FFF) 3D printers’ operators. At the same time, the capillary diameters (~2.1 and 1.2 mm) of the plastometers’ extruders significantly exceed the diameters (100–800 microns of the FFF-3D-printing devices’ nozzles). Thus, the results of standardized melt flow index (MFI) measurements are not applicable for the direct adjustment of technological parameters in the 3D-model slicing software [60,62].

The structural heterogeneity of the FFF-manufactured prototypes’ surface does not always allow 3D-printing products to fully achieve the required strength, tribotechnicality, and other characteristics [63,64] (although the ingredient filling often improves a number of polymer properties [63,64,65,66,67]). Some surface properties [68,69,70] can be achieved with microtexturing techniques (for example, due to the substrate mechanical deformation followed by surface modification [71,72] of the polymer material with fluorine- and oxygen-containing gas mixtures [73,74]). So, the gas-phase treatment provides chemo- and textural-morphological transformations and makes it possible to hydrophobize or hydrophilize the surface to change its antifriction properties, etc. [75,76].

Within the framework of this study, the following were carried out: (a) the biomimetic design [77], (b) the direct regulation of the extrusion technological parameters [74], (c) the composite filaments development, and (d) the manufacture of the 3D-printing products with the increased wear resistance.

## 2. Materials and Methods

### 2.1. The Experimental Samples’ Surface Design

**Figure 1:**
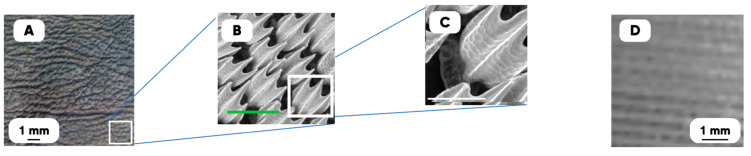
The examples of multi-scale optical (**A**,**D**) and SEM-images (**B**,**C**) of the shark skin texture surface [78] and the surface of a 3D-printed product (**D**). Green scale bars, 200 μm (**B**); white scale bars, 100 μm (**C**) [78].

The design of the experimental samples’ surfaces were carried out using an original technique of the biomimetic approach implementing [77]. The multi-scale images (Figure 1A–C) (obtained with the scanning electron microscopy (SEM) technique (JSM-7500 FA auto emission scanning electron microscope (JEOL, Akishima-sh, Japan)) of the shark skin [78] were chosen as the original layout (due to its well-known, unique hydrotribological properties).

The shark skin structure’s separate element (the “flake”, Figure 1C) is characterized by such a combination of small size and complex shape that it does not allow the correct imitation of the original layout with FFF-3D printing (due to the current FFF technology’s resolution). But the complex (Figure 1B) of flakes forms the mesoscale quasi-periodic structure (Figure 1A) with some similarity to periodic system of 3D strokes of the cured filament (Figure 1D).

The need to form the quasi-periodic structure (similar to the shark skin texture’s mesoscopic mode) on the samples’ surfaces (Figure 1B) were taken into account (to implement the additive prototyping products’ 3D design), and there, the following were carried out:

(1) The formation of the shark skin’s surface digital twin (using the original jpg2xls converter);
(2) The simulation of a set of pixels’ brightness (related to the features of the surface microrelief) with the two-dimensional Fourier series biharmonics decomposing [79,80];
(3) The digital construction of the 3D-printing products structure (based on the obtained model and using Paint3D and Cura slicer software).

The digital image $F\left(x,y\right)$ of the shark skin mesoscopic relief (determining the filament layers’ distribution) was formed as a superposition of the material lattices $B_{kl}\left(x,y\right)$,with $\lambda_{x,y}$ spatial period biharmonics $f_{kl}\left(x,y\right)$ decomposed into a two-dimensional Fourier series:

(1) $$F\left(x,y\right)=\sum_{k=0}^{K}\sum_{l=0}^{L}B_{kl}\left(x,y\right),$$

(2) $$B_{kl}\left(x,y\right)=\left({\vec{b}}_{kl};{\vec{f}}_{kl}\left(x,y\right)\right)=\sum_{m=1}^{4}b_{kl,m}\cdot f_{kl,m}\left(x,y\right),$$

(3) $${\vec{b}}_{kl}^{T}=\left(\begin{array}{c} \begin{array}{c} b_{kl,1}\\ b_{kl,2} \end{array}\\ \begin{array}{c} b_{kl,3}\\ b_{kl,4} \end{array} \end{array}\right)=\frac{1}{N_{kl}}\cdot \iint_{-\infty }^{+\infty }\left\{F\left(x,y\right)\cdot \left(\begin{array}{c} \begin{array}{c} f_{kl,1}\left(x,y\right)\\ f_{kl,2}\left(x,y\right) \end{array}\\ \begin{array}{c} f_{kl,3}\left(x,y\right)\\ f_{kl,4}\left(x,y\right) \end{array} \end{array}\right)\right\}dxdy,$$

(4) $${\vec{f}}_{kl}\left(x,y\right)=\left(\begin{array}{c} \begin{array}{c} f_{kl,1}\left(x,y\right)\\ f_{kl,2}\left(x,y\right) \end{array}\\ \begin{array}{c} f_{kl,3}\left(x,y\right)\\ f_{kl,4}\left(x,y\right) \end{array} \end{array}\right)=\left(\begin{array}{c} \begin{array}{c} \cos\left(2\pi \cdot k\cdot x/\lambda_{x}\right)\cdot \cos\left(2\pi \cdot l\cdot y/\lambda_{y}\right)\\ \cos\left(2\pi \cdot k\cdot x/\lambda_{x}\right)\cdot \sin\left(2\pi \cdot l\cdot y/\lambda_{y}\right) \end{array}\\ \begin{array}{c} \sin\left(2\pi \cdot k\cdot x/\lambda_{x}\right)\cdot \cos\left(2\pi \cdot l\cdot y/\lambda_{y}\right)\\ \sin\left(2\pi \cdot k\cdot x/\lambda_{x}\right)\cdot \sin\left(2\pi \cdot l\cdot y/\lambda_{y}\right) \end{array} \end{array}\right)$$

Their amplitudes $A_{kl}=\sqrt{\sum_{m=1}^{4}b_{kl,m}^{2}}$ set the approximate morphological spectrum of the simulated surface [79,80,81].

**Figure 2:**
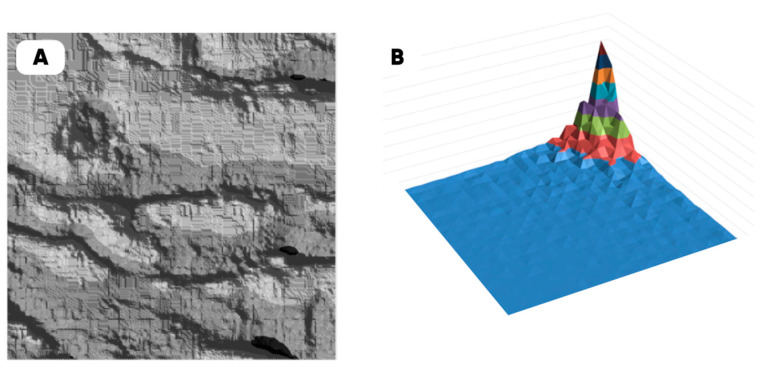
Digital twin (**A**) of the shark skin structural domain and the corresponding morphological spectrum (**B**). The real size of the digital twin square (**A**) is 20 mkm. The indices of the biharmonics are deposited on the axes in the horizontal plane (from 0 to 32); the amplitudes of the biharmonics of the morphological spectrum are deposited on the vertical axis (from 0 to 12).

The characteristic dimension of the shark skin microscale “structural domain” (a fragment of the surface has the same structure and properties as the entire sample “as a whole”) was found using the original technique of variation–rotation patterns [82]. The resulting digital twin of the structural domain and the corresponding morphological spectrum are shown in Figure 2.

The Pearson correlation coefficient was used to quantify the degree of similarity between the digital twins and between the morphological spectra of the original layout and the experimental samples’ surfaces.

(5) $$R_{xy}=\frac{\sum_{k=1}^{N}\left(x_{k}-\bar{x}\right)\cdot \left(y_{k}-\bar{y}\right)}{\sqrt{\sum_{k=1}^{N}{\left(x_{k}-\bar{x}\right)}^{2}\cdot \sum_{k=1}^{N}{\left(y_{k}-\bar{y}\right)}^{2}}}$$

Here, $\left\{x_{k}\right\},\left\{y_{k}\right\}$ are the sets of values of identical information-logical entities characterizing the “original layout” (shark skin) and the “3D-impression” (sample surface).

### 2.2. The Experimental Samples’ Creation

The following were carried out for the 3D-printing test items’ (3D disks) prototyping:

(1) The initial polymer matrix and the bulk modifies the materials’ rational choice;
(2) The polymer matrix dispersed filling with the reinforcing ingredients;
(3) The FFF-technology using prototypes production;
(4) The direct regulation of the sample’s surface functional properties (by the original technique of the fluorine-containing gas mixture modification) [62].

The dominant FFF-3D-printing materials are as follows: acrylic, butadiene nitrile and styrene copolymer (ABS), polylactide (PLA), polyethylene terephthalate glycol (PETG), and thermoplastic polyurethane (TPU) [83,84,85,86,87]. And it was shown in [78] that the PETG-samples’ surfaces have some mesoscopic scale similarity with the shark skin texture in terms of morphological heterogeneity [73].

The dispersed filling [62] with the shungite (NPC Carbon-Shungite LLC, Petrozavodsk, Russia), molybdenum disulfide, montmorillonite (Cloisite 20A, Louisville, KY, USA), carbon functional fibers, and taunite and graphite nanoplates (NPGs) (Well Chem (group) Ind. Co.,Ltd., Quzhou China), whose techniques were used to offer the possibility of the composite filament structure and properties direct regulation. The submitted list of the ingredients is determined by the previously obtained positive results in the ultrahigh molecular weight polyethylene bulk structuring [75].

**Figure 3:**
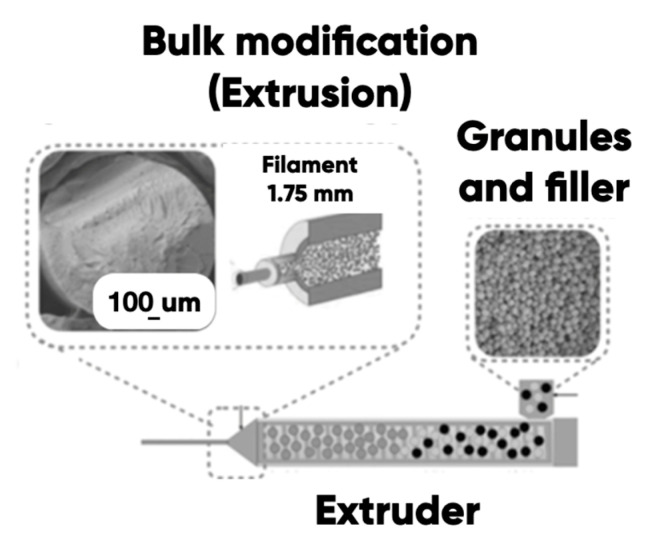
The scheme of the PETG-based composite filament extrusion [89].

All variants of the composite filaments were formed in the single-screw extruder (Filastruder, Twinsburg, OH, USA), the EPS 20 × 25 extrusion line (Polymermashservice, Kuznetsk, Russia), and the modernized twin-screw extruder (Moscow Polytech, Mashplast, Russia) at a temperature of 240 °C in accordance with [62,88] (Figure 3). The melt flow index (MFI) for each obtained filament was measured (see Section 2.3 of Materials And Methods) and the “Flow” parameter was adjusted in the Cura-slicing (3D-rasterization) software (v.5.5).

**Figure 4:**
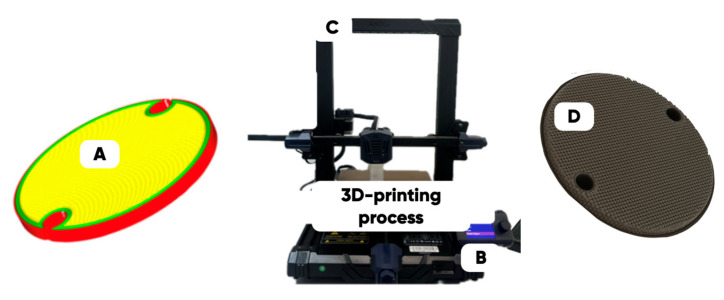
The 3D-printing technology scheme: the digital original 3D-disk layout (made using by Cura) (**A**), the 3D-printing process settings (**B**); the FFF additive manufacturing equipment (**C**) and the image of a manufactured 3D-printing test product (3D disk) (**D**).

A series of the experimental samples (designed for the optimized 3D rasterization conditions in the 3D disks with a diameter of 40 mm and a thickness of 2.5 mm form-factor (Figure 4)) was manufactured on the Anycubic Kobra Go FFF-3D printer (Anycubic, Shenzhen, China) at a temperature of 240 °C and a printing speed of 20 to 40 mm/s (depending on the type and the concentration of the functional filler in the PETG-matrix).

**Figure 5:**
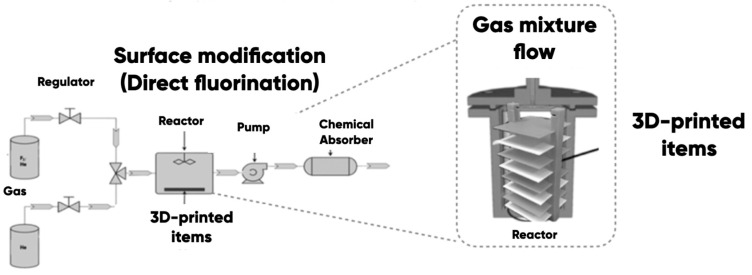
The scheme of 3D-printing products surface fluorine containing gas mixtures modification technique.

The prototypes’ surface properties’ direct control was carried out (in accordance with the materials in the science-structural-functional paradigm) by changing the elemental composition and chemical and physical structures with the helium–fluorine gas mixtures’ (He/F_2_ = X/Y vol.%) treatment at a temperature of 25 ± 2 °C (Figure 5). The high-purity helium (grade 5.0, TU 20.11.11-001-37924839-2019 (99.999%)) and the industrial fluorine (with a purity of at least 99.5%—a residual oxygen content of not more than 0.5%) were used. The modifying gas mixture was prepared by mixing the fluorine and helium in He/F_2_ = 86.5/13.5 vol.% ratio.

The samples’ surfaces were cleaned of potential contamination by a mechanical–chemical method (wiping with ethyl alcohol and drying under normal conditions). The samples were placed into the reaction chamber and were pre-degassed by vacuuming. The He/F_2_ = 86.5/13.5 vol.% gas mixture was supplied to the modification reactor. The chamber was filled with a modifying gas mixture for ~60 s, after which the gas flow was stopped. The atmospheric pressure and the room temperature were in the reactor during the gas-phase modification procedure. The fluorine’s partial pressure was ~0.14·10^5^ Pa. The distance from the reactor entrance to the nearest of the samples was ~5 cm, and to the furthest, ~15 cm. We experimentally established that this distance did not have a statistically significant effect on the efficiency and the quality of the fluorination (due to gas-flow distributor use). The samples were processed within 60 min in accordance with the research program. Then, the reaction products were removed (by vacuuming) with their further decontamination using standard chemical absorbers (CaO). The experimental samples were taken off the reactor chamber and examined empirically. A distinctive feature of the developed and manufactured polymer modification equipment was as follows: (a) the use of 300 cm^3^ stainless steel cylindrical reactor with a gas mixture flow distributor (it provides the entire sample area modification uniformity); (b) the ability to modify the polymer-made items of any geometric shape (varying only the reactor chamber volume) at room temperature without catalyst use [90].

### 2.3. Identifying the Properties and Structure of the Experimental Samples

The following operations were performed to identify the properties and the structure of the composite materials and the 3D-printing products:

(1) The determination of 3D-printing and technological properties of the composite filaments (using the original melt flow index measurement (MFI) technique [62]);
(2) The empirical characterization of the elemental composition and the physical structure of the experimental sample’s surface with scanning electron microscopy (SEM);
(3) The study of tribotechnical properties (the coefficient of friction, the temperature in the tribological contact zone, the wear resistance) of the manufactured products.

**Figure 6:**
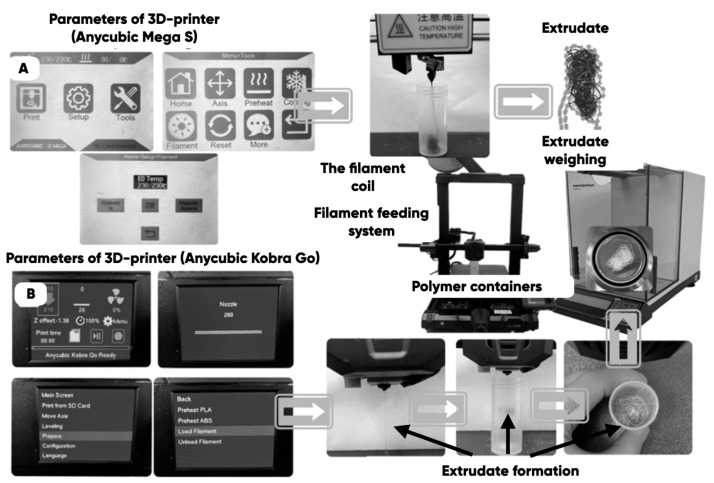
The empirical determination of the actual value of the MFI-3D (g/min) of the developed composite filaments using the Anycubic Mega S (Home >> Tools >> Filament >> Filament In) (**A**) and Anycubric Kobra Go (Menu >> Temperature Nozzle >> Prepare >> Load Filament >> Filament In) (**B**).

The original technique [62] of measuring the composite filaments’ melt flow index (MFI) was used to empirically determine the actual (taking into account the diameters of the extrusion nozzles of a 3D printer) MFI (g/min) of the PETG-based composites. The extrusion was carried out for 60 s through the nozzles of the industrial 3D printers Anycubic Mega S (Figure 6A) and Anycubric Kobra Go (Anycubic, Shenzhen, China) (Figure 6B) with the subsequent weighing of the extrudate on the analytical scales (ViBRA HT-224RCE (SHINKO DENSHI Co., Ltd., Tokyo, Japan); 0.0001 g accuracy).

The value of the “Flow” parameter in the Cura slicer was adjusted as the result of the following MFI-measurements (6):

(6) $${Flow}_{Cura}=\left(1-{MFI}_{com}/{MFI}_{ini}\right)\times 100\%$$

where ${MFI}_{com}$ and ${MFI}_{ini}$ are the melt flow indexes (g/min) of the composition and the initial PETG-based filament.

**Figure 7:**
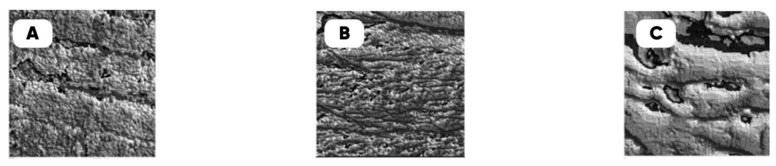
The digital twins of the typical experimental samples’ surfaces: PETG (**A**), PETG fluorinated (**B**), filled with molybdenum disulfide PETG fluorinated (**C**). The real size of the digital twin squares (**A**–**C**) are 20 µm.

The microrelief of the chipped composite filaments and the surface of the 3D-printed products were studied using a JSM-7500 FA autoemission scanning electron microscope (JEOL, Akishima-sh, Japan) at an electron current of ~1 nA. The average depth of analysis (calculated in the Win Casino v2.48 program using the Monte Carlo method) was 0.4 microns. The digital counterparts of the obtained SEM images of the experimental samples’ surfaces are shown in Figure 7.

**Figure 8:**
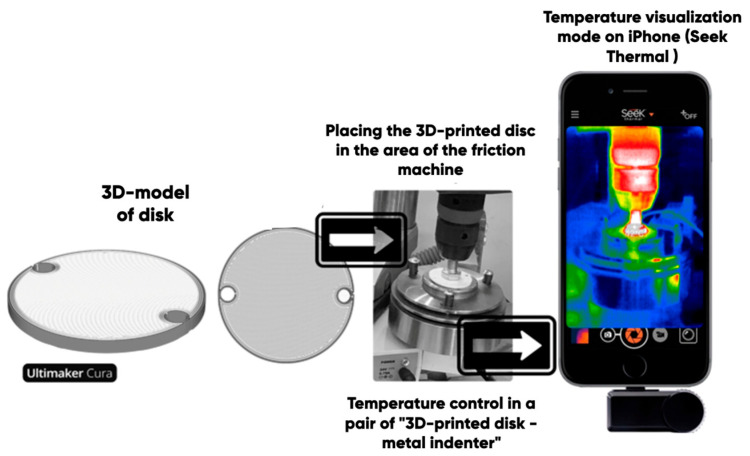
The scheme of the tribological testing procedure (friction coefficient, wear and temperature in the “3D-disk-indenter” contact zone measurements).

The tribotechnical properties (friction coefficient and wear resistance) of the experimental samples (3D disks) were determined with a universal friction machine MTU-01 (JSC Concern Nanoindustria, Saint Petersburg, Russia), with the technique developed at Moscow Polytechnic University [62] in accordance with TU 4271-001-29034600-2004 (in the “dry friction” mode in a pair of “3D-printed disk-indenter”). An annular steel indenter (Ø ~ 10 mm) (rotating at angular speed of 200 rounds per minute) was pressed to the surface of the experimental sample with a force of 147 N (Figure 8). The tribological tests were carried out in an air atmosphere under normal conditions.

The temperature in the “3D-disk-indenter” contact zone was measured using the Seek Thermal Compact Pro thermal imaging module for smartphones. The wear of the experimental samples was determined with the composite 3D-disk mass, reducing gravimetric measurements (ViBRA HT-224RCE analytical scales (SHINKO DENSHI Co., Ltd., Tokyo Japan)) under the standard abrasion (TU 4271-001-29034600-2004) for an hour.

## 3. Results and Discussion

### 3.1. The Results of 3D-Printing Products’ Surface Biomimetic Design

The design of biosimilar material systems [84,91,92,93], the digital twins of the surface (DTS), the variation–rotation pictures, and the surface morphological spectra (SMS) of the shark skin (the original layout) were made in accordance with the original approaches [80,84,94,95]. A quantitative analysis of the variation–rotation pictures allowed us to estimate the characteristic size of the designed product’s surface structural domain and to implement a rational choice of the filament material—PETG (due to its shark skin structure similarity).

The experimental samples were manufactured from PETG filament and based on its composite materials. The DTS, SMS, and values of the Pearson correlation coefficients between DTS/SMS of the original layout (shark skin) and the experimental samples (3D disks) were calculated (Table 1).

It can be seen that the PETG- and PETG-based compositions made experimental samples’ digital twins differ significantly from the shark skin ones ($\left|R_{DTS}\right|<0.1$). However, the obtained surface morphological spectra sufficiently resemble each other ($\left|R_{SMS}\right|>0.9$). The calculated values of the Pearson correlation coefficient show that the fluorinated PETG + MoS_2_ prototypes’ surface mode structures are closer to the shark skin one according to the criterion of the surface morphological spectra similarity.

**Figure 9:**
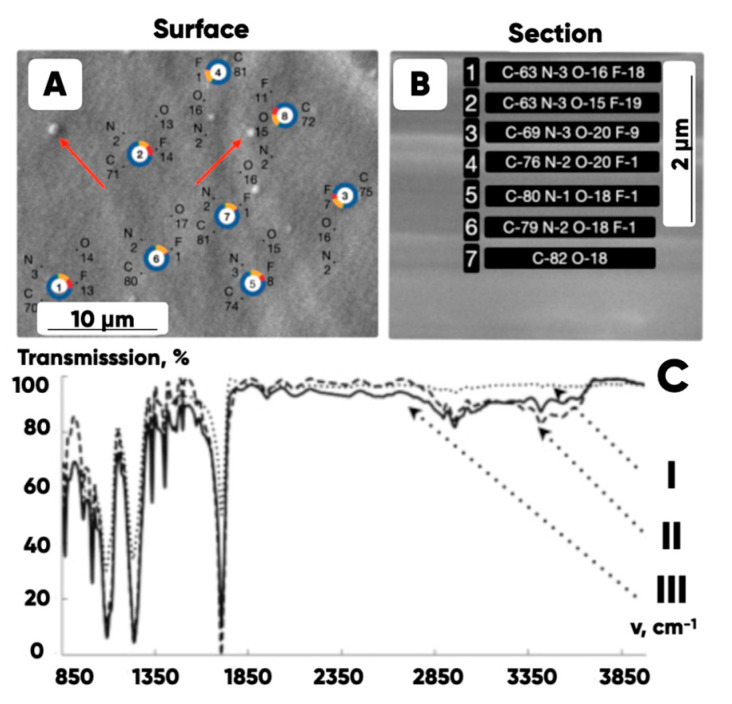
The SEM-images of the top surface (**A**) and the cross-section (**B**) of the oxyfluorinated PET substrate. The numbers indicate the EDS-analysis zones and the elemental composition in atomic % is given. The arrow shows the convex protrusions formed as a result of the modification. The IR spectra (**C**) of the original (I) and the oxyfluorinated PET substrates for 5 (II) and 180 min (III) [96].

The results of the initial and fluorinated PET samples’ IR-Fourier spectroscopy and planar and depth point EDS analysis were presented in [96] (Figure 9).

**Figure 10:**
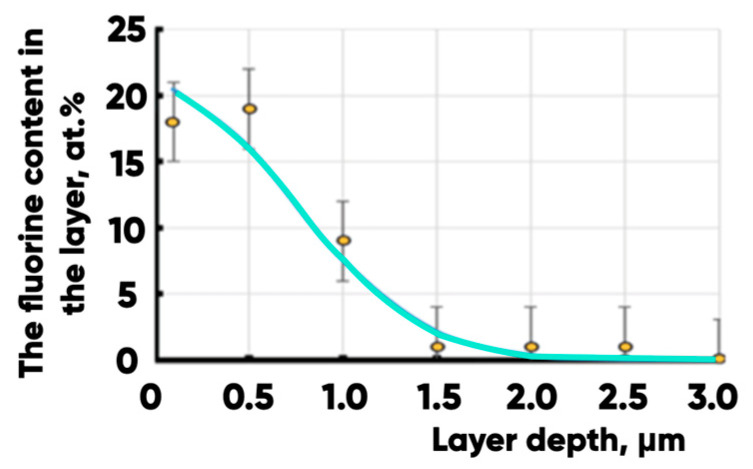
The dependence of the fluorine content on the depth of the modified layer in PETG.

The obtained dependence of the fluorine content on the depth of the layer is shown in Figure 10.

**Figure 11:**
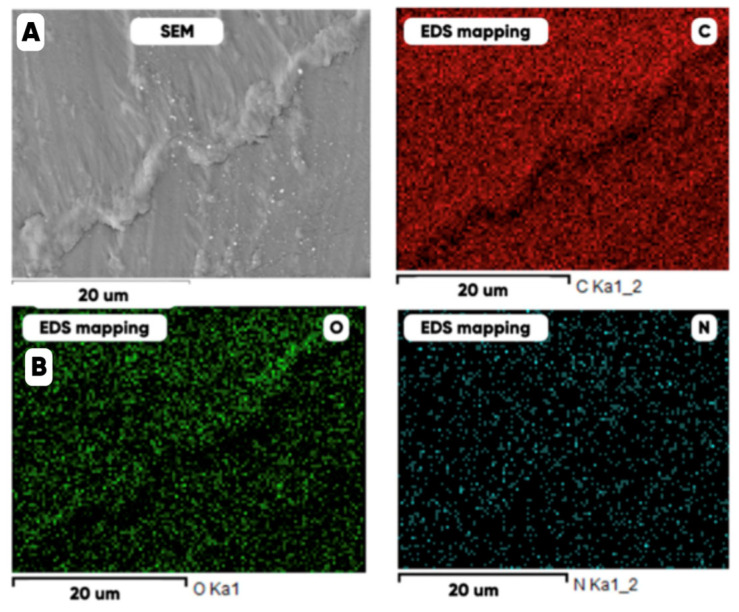
The SEM (**A**) and EDS (**B**) images and the distribution of carbon (C), oxygen (O), and nitrogen (N) over the surface of a PETG filament containing 1 mass.% of thermochromic microcapsules [88].

Similar results were obtained for the typical cured PETG-surface [88] where microrelief, carbon, and oxygen planar distributions were shown (Figure 11).

### 3.2. The Results of the Determined and Developed Composite Filaments’ 3D-Printing Properties

**Figure 12:**
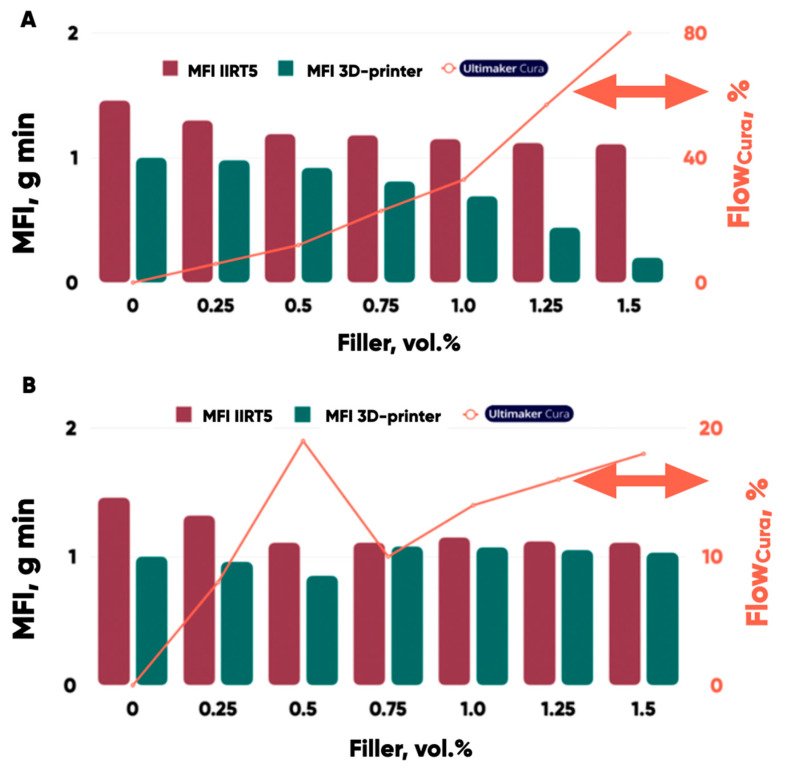
The melt flow indexes (MFIs) of the PETG-based composite filaments filled with shungite (**A**) and molybdenum disulfide (**B**) depending on the MFI-measurement technique—using a plastometer (red) or 3D-printer (green) extruders; orange line (Cura slicer Flow correction parameter in %).

It was found (Figure 12) that the extrusion rate of all produced composite filaments through a 3D-printer nozzle (<500 microns in diameter) significantly differs from the values that can be predicted using the results of industrial plastometer with capillaries 2.1 and 1.2 mm in diameter, in accordance with ASTM 1238 and/or GOST 11645-2021.

The PETG-based composite MFI values are practically independent of the composition and the content of the filler in the polymer matrix when measuring with the IIRT5 industrial plastometer (Figure 12). But the actual MFI values measured using a 3D-printer extruder are significantly lower in some cases! Moreover, the empirical dependence of the composite MFI on the filler concentration is expressed (with a change in the mass fraction of shungite in the range from 0 to 1.5 vol.%). It corresponds to the cases of PETG-based composite filaments filled with the graphite nanoplates (GNP) and taunite (Table 2).

The decrease in the MFI values of the composite filaments occurs mainly due to the formation of the agglomerates (100–400 microns) as a result of the material meso- and microstructure-associated transformations. It worsens the melt passing through the small-diameter 3D-printer extruder nozzle (<500 microns), influencing the quality of the fused filament-fabricated 3D disks negatively [62]. The elimination of the 3D-printing defects (corresponding to correct MFI measurement results) by the Cura slicer (regardless of the software version) technological extrusion parameter (“Flow”) made it possible to form the quasicontinuous material systems (3D disk) from the PETG-based (filled with the MoS_2_, shungite, and other ingredients) composite filament layers.

### 3.3. The Results of the Formed Integrated Fluorine-Containing Layers’ Tribological Properties’ Determination

**Figure 13:**
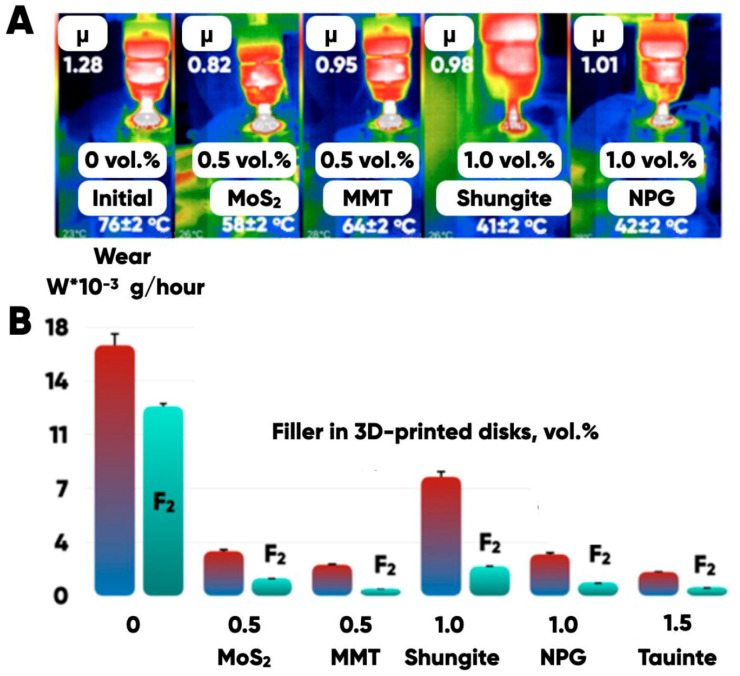
The temperature in the “indenter-3D-printed disk” contact zone and the friction coefficients (**A**) and wear (**B**) of the experimental samples made on the basis of PETG (initial; filled with molybdenum disulfide, montmorillonite, shungite, graphite nanoplates, taunite; fluorinated).

It is shown (using direct measurements of the friction coefficient, wear, and temperature in the tribological contact zone (“indenter-3D-printed disk”) of manufactured samples (based on PETG filled with the shungite, montmorillonite, graphite nanoplates and molybdenum disulfide)) that the chemo-morphological texturing with the fluorine-containing gas mixtures provides the ability to control the tribotechnical characteristics of the additive prototyping products’ properties (Figure 13).

Additionally, we have calculated the specific wear rate $k\left(\frac{m\cdot s}{kg}\right)$ [97]:

(7) $$k=\frac{V}{F\cdot L}=\frac{M}{\rho_{PETG}\cdot F\cdot L}$$

Here, V and M are wear products’ volumes and masses; $\rho_{PETG}≅1270\left(\frac{kg}{m^{3}}\right)$—the PETG density; $F≅147\left(N\right)$—the normal force load; L—the equivalent wear length: $L=2\pi \cdot r\cdot N≅71\left(m\right)$; $N=\nu \cdot \tau =200\left(\frac{round}{minute}\right)\cdot 15\left(min\right)=3000\left(rounds\right)$—the number of wear cycles; $r≅3.75\left(mm\right)$—the annual indenter contact average radius; $\nu =200\left(\frac{round}{minute}\right)$ and $\tau =15\left(min\right)$—the rotation speed and the wear test duration, respectively. The calculation results are presented in Table 3.

We tried to determine the empirical dependence of the samples’ actual wear on the rest friction coefficient. The latter was measured using the universal friction machine MTU-01 in accordance with TU 4271-001-29034600-2004 (in the “dry friction” mode in a pair of “3D-printed disk-indenter”) (Figure 8). The measured average values and standard deviations of the rest friction coefficients for a number of PETG-based samples are shown in Table 4.

The classical Reye–Archard–Khrushchev law of wear (which formalizes the description of the energy dissipation hypothesis) has the following form:

(8) $$V=K\cdot \frac{N\cdot L}{H}$$

Here, V is the total volume of wear products; K—a dimensionless constant; N—the total normal load; L—the sliding distance; H—the hardness of the material.

We directly measured the rate of the weight loss per time. So, the above dependence was transformed to the following form:

(9) $$w=\frac{∆m}{∆t}=K\cdot \rho \cdot \frac{N\cdot v}{H}$$

Here, ρ is the density of the wear product material; v=ω·r is the average linear velocity of the indenter surface element r—located from the rotation axis. The only value that actually changed during the abrasive wear process in (9) was the hardness of the sample surface. It is known [98,99,100] that the hardness, as a rule, decreases non-linearly with the temperature increasing. Since there is no universal model of the hardness on temperature dependence, it can be approximated by monotonically decreasing biparametric power-law $H=\alpha \cdot T^{-\beta }$ or exponential $H={\alpha \cdot e}^{-\beta \cdot T}$ functions (at first approximation). In this case, the average temperature in the tribological contact zone linearly depends on friction force, which, in turn, linearly depends on the friction coefficient (under the constant load and the same test duration): $T\sim F_{TP}\cdot L\sim \mu \cdot N$. Thus, the following formulas are presented:

(10) $$w=\frac{∆m}{∆t}\sim K\cdot \rho \cdot \frac{N\cdot v}{\alpha \cdot e^{-\beta \cdot T}}\sim a\cdot e^{b\cdot \mu }$$

or

(11) $$w=\frac{∆m}{∆t}\sim K\cdot \rho \cdot \frac{N\cdot v}{\alpha \cdot T^{-\beta }}\sim a\cdot \mu^{b}$$

It also mentioned [101] that the hardness of polymer materials can increasingly weaken as the low temperature grows. Thus, the hardness on temperature dependence should be extreme and can be approximated by the parabolic general form $H=\alpha \cdot T^{2}+\beta \cdot T+\gamma$ with α<0. A possible form of this type dependencies’ parameterization is also $H=\beta -\alpha \cdot {\left(T-T_{0}\right)}^{2}$. In this case, the following formula applies:

(12) $$w\sim K\cdot \rho \cdot \frac{N\cdot v}{\alpha \cdot \mu^{2}+\beta \cdot \mu +\gamma }\sim \frac{1}{b-a\cdot {\left(\mu -\mu_{0}\right)}^{2}}$$

We used the simplified biparametric superhyperbolic model for the unification and to ease the comparison with other models:

(13) $$w\sim K\cdot \rho \cdot \frac{N\cdot v}{{\left(\alpha \cdot T+\beta \right)}^{2}}\sim \frac{1}{{\left(\alpha \cdot \mu +\beta \right)}^{2}}$$

This allowed us to compare the quality of the same data set approximation by different models, calculating the classical determination coefficient:

(14) $$R^{2}=1-\frac{\sum_{i=1}^{N}\varepsilon_{i}^{2}}{\sum_{i=1}^{N}{\left(w_{i}-\bar{w}\right)}^{2}}$$

Here, N is the data set element’s number; $\left\{\varepsilon_{i}^{2}\right\}$—the residual squares; $\bar{w}$—the average value of measured ${\left\{{\left(∆m/∆t\right)}_{i}\right\}}_{N}$.

We constructed the confidence intervals at $\left(w\pm 2\cdot \delta w\right)$ and $\left(w\pm 3\cdot \delta w\right)$ levels, where δw was determined by the following differential technique:

(15) $$w=F\left(\mu ,a,b\right)\Rightarrow \delta w=\left|{\left.\frac{\partial F}{\partial \mu }\right|}_{\bar{a},\bar{b}}\cdot \bar{\delta \mu }\right|+\left|{\left.\frac{\partial F}{\partial a}\right|}_{\bar{\mu },\bar{b}}\cdot \bar{\delta a}\right|+\left|{\left.\frac{\partial F}{\partial b}\right|}_{\bar{a},\bar{\mu }}\cdot \bar{\delta b}\right|$$

The corresponding linearized models’ parameters and their standard deviations were determined using the least squares technique.

**Figure 14:**
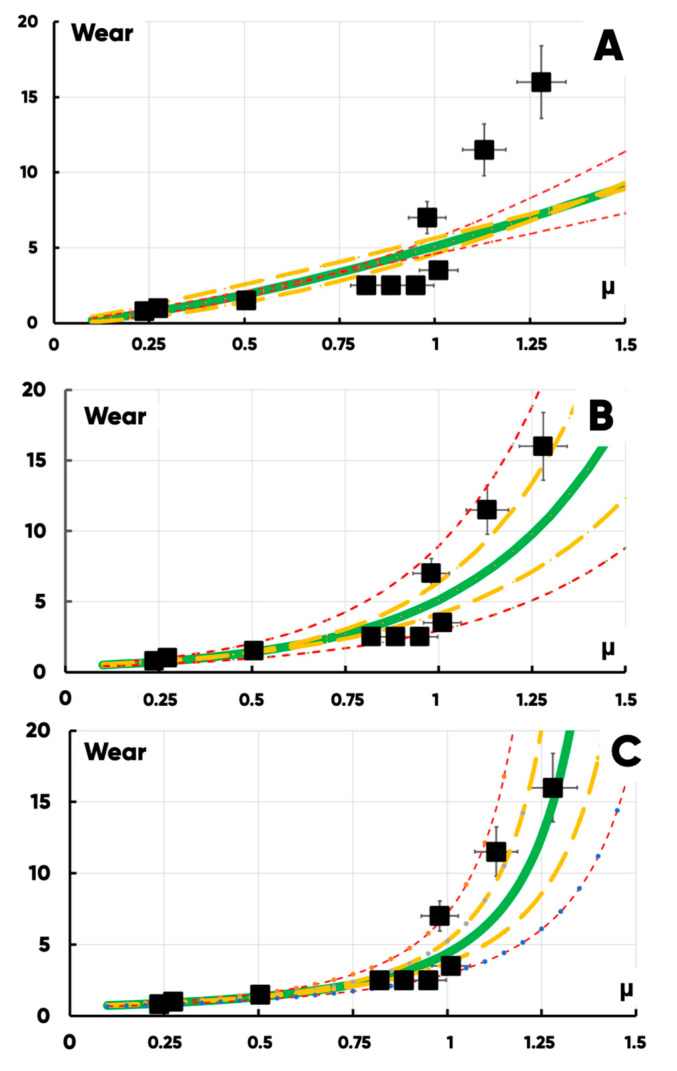
The dependences (power-law (**A**), exponential (**B**), and superhyperbolic (**C**)) of wear on the friction coefficient for the all types of experimental samples. The dots are the average values of the measurement results; the green lines are approximating functions; the yellow and red lines are confidence intervals for the models (at levels 0.95 and 0.99, respectively).

It was found (based on a comparison of the approximation quality of the experimental data set with some analytical functions) that a generalized description of the product surface wear on the rest friction coefficient dependence (for the “indenter-3D-printed disk” tribological contact pair) should be carried out using a second-order superhyperbolic model (Figure 14, Table 5).

The necessity of using this model type is also supported by the presence of a vertical asymptote that corresponds with the limit friction coefficient value $\mu_{critical}$, at which the destruction of the hypothetical (with $\mu >\mu_{critical}$) PETG-based sample could occur as a result of the tribological interactions (it does not happen in practice because we try to use modification methods to reduce the friction and the wear rather than to increase it).

A significant (non-linear) reduction in wear (Figure 14) can be achieved by small changes in the “large” rest friction coefficient and decreases in the “low” one, which is not so sufficient for PETG-composite samples’ wear-resistance growth. We separated the effects of bulk (filling) and surface (fluorination) modifications by testing the wear of PETG, PETG + filler (without fluorination), PETG (with fluorination) and PETG + filler with fluorination samples under the same conditions (Table 6). All measurements were performed for a 10-sample series with the identical technological history and statistical data-processing procedures.

The proposed complex technology for increasing wear resistance has demonstrated the greatest effectiveness for taunite and montmorillonite-filling PETG-based items. The highest relative fluorination efficiency was observed as a result of gas-phase modification of the PETG/montmorillonite samples.

The surface treatment with the fluorine-containing gas mixtures significantly increases the wear resistance of the prototypes made from both raw and filled PETG. The bulk modification of the filament with molybdenum disulfide (0.5 vol.%), MMT, NPG and taunite reduces the composites’ wear by four to six times compared with an unfilled case. And the fluorination additionally reduces the composites’ wear up to three times due to the formation of an integrated fluoroplast-like layer with a depth of ~2 microns in the composite samples’ surfaces (Figure 9).

A decrease in the average temperature (from 78 ± 2 to 42 ± 2 °C) of 3D-printed disks in the tribological contact zone (Figure 13) confirms a decrease in the adhesion force in the “indenter-3D-printed disk” pair that is due to the morphological (structural and chemical) transformations of the modified surface.

We present the results of standardized (ISO 25178-2:2012) three-dimensional surface parameters’ measurements (Table 7). According to ISO, $S_{a}$ is the arithmetic mean height of the scale limited surface; $S_{q}$ is the root mean square height of the scale-limited surface; $S_{sk}$ is the skewness of the scalelimited surface; $S_{ku}$ is the kurtosis of the scale-limited surface; $S_{al}$ is the autocorrelation length; and $S_{tr}$ is the texture aspect ratio. We calculated $S_{al}$ and $S_{tr}$ at the 0.5 level. Each measurement was performed for a group of 10 samples with identical technological history. Table 7 shows the corresponding average values and errors for PETG–based samples—initial, fluorinated, and montmorillonite bulk-modified and -fluorinated.

The statistically significant differences in the above parameters’ values are observed exclusively for the $S_{al}$ characteristic. Taking into account the radical differences in the wear of the considered samples, they indicate the determining role of induced changes in the roughness mode structure by the modification. And it removes the degeneracy of the statistically indistinguishable surface heterogeneity characteristics conditioned by their excessive generalization.

The morphological heterogeneity of the experimental samples’ surface was characterized quantitatively as a result of the computer analysis of the corresponding optical images (Table 8) after the completion of the tribological tests. When analyzing the corresponding images, we used the morphological spectra technique ((1)–(4); Figure 2 [79,94,95]), which is in some ways is analogous to power spectral density (PSD) calculations.

It can be seen (Table 8) that the maximum mean-square values of the morphological spectrum biharmonic amplitudes ($MSV=\sqrt{\sum_{kl}A_{kl}^{2}}=0.055$) were established for optical images of the samples obtained as a result of the fluorination of 3D disks made from the PETG-based composite filaments containing 0.5 vol.% molybdenum disulfide. And the minimum ($\sqrt{\sum_{kl}A_{kl}^{2}}=0.023$) one was for a sample containing 1 vol.% $MoS_{2}$.

An increase in the content of molybdenum disulfide (from 0.5 to 1.5 vol.%) in the polymer matrix helps to preserve the initial quasi-periodic surface texture of the 3D-printed disk (since $\sqrt{\sum_{kl}A_{kl}^{2}}$ grows). At the same time, the relative fluorination efficiency reduces with an increase in the volume fraction of $MoS_{2}$ in the PETG matrix ($\sqrt{\sum_{kl}A_{kl}^{2}}$ shrinks). This is probably due to the specific transformations of the supramolecular structure of the composite caused by the dispersed filling (a change in the ratio of the amorphous and crystalline phases of the polymer matrix, an increase in the molar volume of the surface layer, a chemisorption of the active reagent by $MoS_{2}$ agglomerates, etc.).

Thus, the consistent use of bulk and surface modification makes it possible to effectively regulate the tribotechnical properties of 3D-prototyping products developed on the basis of the composite filaments.

## 4. Conclusions

Taking into account the biomimetically determined need to ensure the similarity of the 3D-printed items’ surface structures to the shark skin texture (to improve their tribological characteristics) and apply the original approach to the rational choice of the main extrusion parameter value, we developed a technology for the formation of an antifriction-integrated fluorine-containing coating in the surface layers of the products manufactured with FFF-3D printing from the PETG-based filaments filled with shungite, molybdenum disulfide, montmorillonite, etc.

The possibility of friction coefficient, wear resistance, and temperature in the tribological contact zone’s direct regulation is due to the presence of the reinforcing fillers in the polymer matrix. The changes in their concentration (in the studied range) do not lead to a critical deterioration of the composite filaments’ printing-technical properties (compared to the original unfilled polymers) but allow the frictional properties of the 3D products’ surfaces to improve.

The primary increase in the wear resistance of the 3D-printed disks (due to the filling of the polymer (PETG) matrix) is explained by the mechanical activation of the reinforcing filler (MoS_2_) during the abrasion process. A further reduction in wear is associated with the friction coefficient decreasing (in the “indenter-3D-printed disk” contact pair), achieved as a result of the surface treatment with a fluorine-containing gas mixture.

The chemo-induced decrease in the friction coefficient value is a consequence of a reduction in the products’ surface roughness due to the formation of an integrated coating (up to 2 microns thick), chemically similar to the fluoroplastics.

The following are the future tasks of the research team: the optimization of the gas-phase modification procedure’s technological parameters; the base polymer and the fillers’ granulometric composition, the temperature regime, and the geometry of extrusion influence on the 3D-printing products’ volume structure and studied structural characteristics; and the analysis of the fluorinated surfaces’ stability and the possibility of transition films’ formation under the increased abrasion duration, various loads, and indenter rotational speeds, using start/stop cycles for the analysis of shock tribological resistance, etc.

## Figures and Tables

**Table 1:** The digital twins of the surface (DTS) and the profile projections of the averaged surface morphological spectra (SMS) for the original layout (shark skin) and for the PETG-based experimental samples (3D disks) with the corresponding values of the $R_{DTS}$ and $R_{SMS}$ Pearson correlation coefficients. The real size of the digital twin squares are 20 mkm.

Parameter/Materials	Shark Skin	PETG	PETG + F_2_	PETG + MoS_2_ + F_2_
The digital twin of the surface	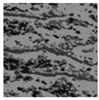	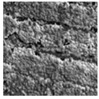	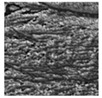	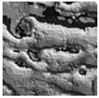
$R_{DTS}$	-	0.06 ± 0.01	0.011 ± 0.006	0.011 ± 0.009
The profile projections of the averaged surface morphological spectra	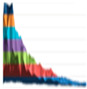	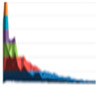	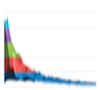	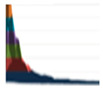
$R_{SMS}$	-	0.915 ± 0.005	0.928 ± 0.005	0.935 ± 0.005

**Table 2:** The melt flow indexes (MFIs) of the PETG-based composite filaments filled with graphite nanoplates and taunite.

Filler Content in the PETG Filament mass.%	MFI_3D printer_ (g/min)		
	0.5	1.0	1.5
GNP	0.884 ± 0.009	0.882 ± 0.009	0.549 ± 0.005
Taunite	0.810 ± 0.008	0.443 ± 0.005	0.065 ± 0.007

**Table 3:** The results of wear coefficient k calculations: $F=147\left(N\right)$; $L≅71\left(m\right)$; $\rho_{PETG}≅1270\left(\frac{kg}{m^{3}}\right)$.

$k=\frac{M}{\rho_{PETG}\cdot F\cdot L}$	PETG	PETG + F2	PETG + MoS2	PETG + MoS2 + F2	PETG + MMT	PETG + MMT + F2	PETG + Shungite	PETG + Shungite + F2	PETG + NPG	PETG + NPG + F2	PETG + Taunite	PETG + Taunite + F2
$\left(\begin{array}{c} M\\ \pm ∆M \end{array}\right)\cdot 10^{6},kg$	4.5 ± 0.4	3.5 ± 0.3	0.15 ± 0.05	0.37 ± 0.08	0.6 ± 0.1	0.13 ± 0.04	2.1 ± 0.2	0.70 ± 0.09	1.1 ± 0.1	0.33 ± 0.05	0.36 ± 0.08	0.20 ± 0.05
$\left(\begin{array}{c} k\\ \pm ∆k \end{array}\right)\cdot 10^{15},\frac{m\cdot s}{kg}$	340 ± 30	270 ± 20	11 ± 3	28 ± 5	48 ± 6	10 ± 3	160 ± 10	53 ± 9	78 ± 10	25 ± 5	27 ± 6	15 ± 4

**Table 4:** The average values and the standard deviations of the samples’ rest friction coefficients.

Rest Friction Coefficient	PETG	PETG + F_2_	PETG + MoS_2_	PETG + MoS_2_ + F_2_	PETG + MMT	PETG + MMT + F_2_	PETG + Snungite	PETG + Snungite + F_2_	PETG + NPG	PETG + NPG + F_2_
μ	1.28	1.13	0.82	0.27	0.95	0.24	0.98	0.9	1.0	0.51
∆μ	0.15	0.15	0.09	0.06	0.09	0.06	0.09	0.1	0.1	0.08

**Table 5:** The results of the approximating functions parameters’ specifications when the wear on the friction coefficient is studied.

MODEL	$W=F\left(\mu \right)$	a±∆a	b±∆b	$R^{2}$
Power-law	$W=a\cdot \mu^{b}$	1.6±0.2	5.1±0.5	0.47
Exponential	$W=a\cdot e^{b\cdot \mu }$	2.6±0.4	0.39±0.07	0.73
Superhyperbolic	$W={\left(a\cdot \mu +b\right)}^{-2}$	−0.78±0.07	1.26±0.06	0.86

**Table 6:** The PETG-based samples’ wear reduction efficiencies.

Wear	Fluorination	PETG	+F_2_		Composition
Filling	Synergy				
PETG		16 ± 2	13 ± 1	−19%	PETG/F_2_
+Shungite		7.5 ± 0.8	2.0 ± 0.5	−73%	PETG/Shungite/F_2_
		−53%	−85%	−88%	
+NPG		3.0 ± 0.5	1.5 ± 0.5	−50%	PETG/NPG/F_2_
		−81%	−88%	−91%	
+MoS2		3.0 ± 0.5	1.0 ± 0.5	−67%	PETG/MoS2/F_2_
		−81%	−92%	−94%	
+Taunite		1.5 ± 0.5	0.5 ± 0.5	−67%	PETG/Taunite/F_2_
		−91%	−88%	−97%	
+MMT		2.0 ± 0.5	0.5 ± 0.5	−75%	PETG/MMT/F_2_
		−88%	−96%	−97%	

**Table 7:** The standardized three-dimensional surface parameters of the of PETG-based samples: initial, fluorinated and MMT-filled and -fluorinated.

Standardized Three-Dimensional Surface Parameters	PETG	PETG + F_2_	PETG + MMT + F_2_
$S_{a}$, µm	0.46 ± 0.01	0.47 ± 0.01	0.48 ± 0.02
$S_{q}$, un.	14.5 ± 0.3	14.9 ± 0.4	15.2 ± 0.7
$S_{sk}$, un.	1.63 ± 0.03	1.61 ± 0.02	1.58 ± 0.07
$S_{ku}$, un.	3.2 ± 0.2	3.1 ± 0.1	3.0 ± 0.3
$S_{al}$, µm	0.24 ± 0.04	0.16 ± 0.02	0.7 ± 0.1
$S_{tr}$, un.	0.7 ± 0.2	0.6 ± 0.3	0.5 ± 0.2

**Table 8:** The morphological spectra of the optical images and mean-square values (MSVs) of the biharmonic amplitudes quantifying the mode structure of the surface roughness for the 3D disks made from PETG-based composite filament bulk, modified with the molybdenum disulfide and surface-modified with a 60 min gas mixture He/F_2_ = 86.5/13.5 vol.% treatment).

Molybdenum Disulfide Content, vol.%	The Morphological Spectra for the Optical Images of the PETG-Based Composite 3D-Disks’ Surface	
	Initial	86.5%He + 13.5%F_2_
0.5	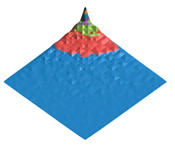 MSV ~ 0.025	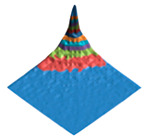 MSV ~ 0.055
1.0	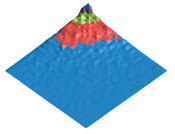 MSV ~ 0.023	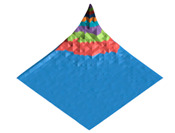 MSV ~ 0.037
1.5	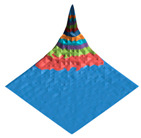 MSV ~ 0.047	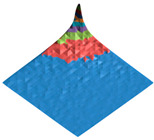 MSV ~ 0.033

## Data Availability

Data are contained within the article.
